# Discordance of Mutation Statuses of Epidermal Growth Factor Receptor and K-ras between Primary Adenocarcinoma of Lung and Brain Metastasis

**DOI:** 10.3390/ijms17040524

**Published:** 2016-04-07

**Authors:** Kun-Ming Rau, Han-Ku Chen, Li-Yen Shiu, Tsai-Ling Chao, Yi-Ping Lo, Chin-Chou Wang, Meng-Chih Lin, Chao-Cheng Huang

**Affiliations:** 1 Division of Hematology-Oncology, Department of Internal Medicine, Kaohsiung 833, Taiwan; kmrau58@adm.cgmh.org.tw; 2 Chang Gung Memorial Hospital, Kaohsiung, Taiwan and Chang Gung University College of Medicine, Tao-Yuan 333, Taiwan; ccwang52@adm.cgmh.org.tw (C.-C.W.); mengchih@adm.cgmh.org.tw (M.-C.L.); 3 Department of Pathology, Yuan’s General Hospital, Kaohsiung 802, Taiwan; chenhanku@gmail.com; 4 Biobank and Tissue Bank, Kaohsiung Chang Gung Memorial Hospital, Kaohsiung 833, Taiwan; her2neu24@gmail.com (L.-Y.S.); monica0977@hotmail.com (Y.-P.L.); 5 Department of Medical Research, Cell Therapy and Research Center, E-Da Hospital, I-shou University, Kaohsiung 840, Taiwan; 6 Department of Laboratory Medicine, Kaohsiung Chang Gung Memorial Hospital, Kaohsiung 833, Taiwan; tsaeling@cgmh.org.tw; 7 Division of Pulmonary and Critical Care Medicine, Department of Internal Medicine, Kaohsiung Chang Gung Memorial Hospital, Kaohsiung 833, Taiwan; 8 Biobank and Tissue Bank and Department of Pathology, Kaohsiung Chang Gung Memorial Hospital, Kaohsiung 833, Taiwan

**Keywords:** adenocarcinoma of lung, EGFR mutation, K-ras mutation, brain metastasis, target therapy

## Abstract

Mutations on epidermal growth factor receptor (EGFR) of adenocarcinomas of lung have been found to be associated with increased sensitivity to EGFR tyrosine kinase inhibitors and K-ras mutations may correlate with primary resistance. We aimed to explore the discordant mutation statuses of EGFR and K-ras between primary tumors and matched brain metastases in adenocarcinomas of lung. We used a sensitive Scorpion ARMS method to analyze EGFR mutation, and Sanger sequencing followed by allele-specific real-time polymerase chain reaction to analyze K-ras mutation. Forty-nine paired tissues with both primary adenocarcinoma of lung and matched brain metastasis were collected. Thirteen patients (26.5%) were discordant for the status of EGFR between primary and metastatic sites. K-ras gene could be checked in paired specimens from 33 patients, thirteen patients (39.6%) were discordant for the status of K-ras. In primary lung adenocarcinoma, there were 14 patients of mutant EGFR had mutant K-ras synchronously. This study revealed that the status of EGFR mutation in lung adenocarcinomas is relatively consistent between primary and metastatic sites compared to K-ras mutation. However, there are still a few cases of adenocarcinoma of lung showing discordance for the status of EGFR mutation. Repeated analysis of EGFR mutation is highly recommended if tissue from metastatic or recurrent site is available for the evaluation of target therapy.

## 1. Introduction

Lung cancer is one of the most common causes of cancer death worldwide [1], and adenocarcinoma is the major subtype [2]. Traditionally, patients with non-small cell lung carcinoma (NSCLC) have been treated with surgical resection with or without adjuvant chemotherapy or palliative systemic therapies for those at advanced stage. However, because of the high mortality rate and the variable treatment responses of lung cancer, a great deal of research has focused on molecular markers of NSCLC that could serve as potential therapeutic targets.

In the past 10 years, the overall survival of lung cancer patients has greatly improved to a median of 12 months and even longer in some clinical studies [3]. This progress is due to the introduction of new drugs and individualized therapy based on different histological subtypes and driver mutations that determine the biology of lung cancers and can be used to predict drug efficacy [4]. The epidermal growth factor receptor (EGFR) gene is currently the most promising and drugable oncogene in NSCLC. Another important predictive and therapeutic biomarker is a fusion tyrosine kinase from the fusion gene of *echinoderm microtubule-associated protein-like 4* (*EML4*) and the *anaplastic lymphoma kinase* [5,6]. Targeting EGFR, especially by using EGFR tyrosine kinase inhibitors (TKIs), has played a central role in advancing NSCLC research, treatment and outcome prediction. Recently, the EGFR TKI had also been proved to improve the overall survival in certain EGFR mutation [7].

Specific EGFR mutations are associated with the sensitivity to EGFR TKIs. Somatic mutations include small in-frame deletions and amino-acid substitutions at the ATP-binding pocket of the tyrosine kinase domain. Small exon 19 deletion (Del19) and exon 21 point mutation (L858R) are the two most common mutations associated with improved outcomes with EGFR TKI therapy [8,9,10]. K-ras is another oncogene, more commonly with mutations in smokers. Compared with an approximate 50% mutation rate of the gene encoding EGFR in Asian patients [11], the mutation rate of EGFR is only 10%–15% in Caucasian populations [12]. In these populations, K-ras is the most commonly mutated oncogene in lung cancers in Western countries, with activating point mutations in 15%–20% of all patients of NSCLC [13,14] and 25%–35% of adenocarcinomas [15,16]. Many studies have suggested that mutated K-ras is associated with a worse overall survival in patients with NSCLC [17]; anti-EGFR therapies are ineffective for K-ras mutant tumors [18,19], which are associated with lack of sensitivity and poorer clinical outcomes when treated with EGFR TKIs or chemotherapy [18,20].

Although the status of EGFR and K-ras mutations has been proposed to guide patient selection for anti-EGFR TKI therapy, the majority of EGFR and K-ras mutations are evaluated only in primary tumors because tumor tissue from the metastatic site is not always available. To date, only a few small-scale studies have analyzed the mutation status of EGFR and K-ras in both primary and metastatic sites of lung cancer [21,22,23]. Since EGFR TKIs are mainly used to treat lung cancer patients with metastatic diseases, differences in EGFR and K-ras mutations between the primary and metastatic sites may influence the outcome of such a therapy. In particular, since intra-tumor heterogeneity is a common phenomenon that may also occur in primary tumors and metastatic sites [24,25], different degrees of therapeutic response at different sites are not rare.

The aim of this study was to compare the statuses of K-ras and EGFR between primary adenocarcinomas of lung and their corresponding brain metastases, and to investigate whether the existence of genetic alterations would influence the outcomes of patients.

## 2. Results

Fifty-seven pairs of paraffin-embedded tissues with both primary adenocarcinoma of lung and matched brain metastases were collected from 1991 to 2010. Eight pairs of specimens were excluded due to poor DNA quality after long-term storage. There were 27 male and 22 female patients. Median age at diagnosis was 63.0 years. The majority of patients (75.5%) were diagnosed with stage IV disease. Thirty-one patients (63.3%) were diagnosed with lung adenocarcinoma synchronous with brain metastasis. Because we included patients before the introduction of EGFR TKIs, there were only 14 patients (28.6%) receiving EGFR TKI treatment (Table 1).

Evaluation of the mutation status of EGFR in primary tumors and brain metastases showed that 30 (61.2%) primary tumors and 30 (61.2%) of their paired brain metastases had EGFR mutations. L858R was the predominant mutation in primary tumors, whereas Del19 was predominant in brain metastases. Thirty-six patients (73.5%) had the same EGFR genotype in both primary and metastatic sites. Four patients with L858R mutation and two patients with Del19 mutation at primary tumors lost these sensitive mutations at brain metastases (Table 2)

Analysis of K-ras mutation was available in 33 pairs of specimens. To increase the detection sensitivity, we used Sanger sequencing to check K-ras gene first, followed by allele-specific real-time polymerase chain reaction (PCR) for cases with no detectable K-ras mutations by Sanger sequencing. Once K-ras mutation was found by Sanger sequencing, the patient would be classified to mutation positive without further PCR detection. Ten patients of primary tumors and 15 patients of brain metastases were wild type (WT) by Sanger sequencing, but they turned out to be mutant K-ras by real-time PCR. Nineteen primary adenocarcinomas and 17 brain metastases of the 33-paired specimens had WT K-ras. Ten patients of primary tumors and 15 patients of brain metastases were WT by Sanger sequencing, but they turned out to be mutant K-ras by real-time PCR. Twenty patients (60.6%) had the same genotype in both primary and metastatic sites, whereas seven patients of WT at primary tumors changed to be mutant K-ras at brain metastases, and five patients of mutant K-ras at primary tumors changed to be WT at brain metastases. Variety of K-ras mutation statuses between primary site and brain metastasis was common (Table 3). In summery, there were 13 patients discordant for the status of EGFR between primary and metastatic sites (Table 2) and 13 patients discordant for the status of K-ras between primary and metastatic sites (Table 3). In these patients, five cases in EGFR and two cases in K-ras were analyzed metachronous and the others were synchronous.

Because K-ras mutation might lead to resistance to EGFR TKIs [26], we then analyzed the mutation status of EGFR and K-ras in primary tumors and brain metastases to see whether the mutation status of K-ras and EGFR differed between primary tumors and matched brain metastases (Table 4). In the total 19 patients whose primary tumors had WT EGFR, 12 also had WT K-ras (63.2%). Fourteen out of 30 patients with EGFR mutations also had K-ras mutations (11 patients with L858R and three with Del19) in primary tumors. In addition, 16 out of 28 patients (57.1%) with WT K-ras had a mutant EGFR in the primary lung adenocarcinoma. These findings were similar in the brain metastases, where seven out of 11 patients (63.6%) with WT EGFR also had WT K-ras, and 10 out of 17 patients (58.8%) with WT K-ras had mutant EGFR in brain metastases. Interestingly, in brain metastases, Del19 was more frequently associated with WT K-ras than L858R was.

Differences in genotypes between the primary tumors and brain metastases are summarized in Table 5. Among 19 patients with WT EGFR in primary tumors, there were four patients (21%) with mutant EGFR in brain metastases. In contrast, among 30 patients with mutant EGFR in primary tumors, there were four patients (13.3%) with WT EGFR in brain metastases. In K-ras status, we found that among 19 patients with WT K-ras in primary tumors, there were seven patients (36.8%) with mutant K-ras in brain metastases. Among 14 patients with mutant K-ras in primary tumors, there were five patients (35.7%) with WT K-ras in brain metastases. Compared with EGFR, K-ras showed a higher discordant rate between primary tumors and brain metastases.

Fourteen patients received EGFR TKIs as a part of their treatments. Ten cases received EGFR TKIs as the secondary line therapy, four cases received EGFR TKIs as the third line therapy and none of our patients received EGFR TKIs as the first line therapy. Treatment responses according to different genotypes are presented in Table 6. One patient who had WT EGFR and K-ras in primary tumor reached a complete response (CR). However, the EGFR mutation in the brain metastasis of the patient was Del19. Four out of six patients who had L858R mutation in primary tumors achieved a partial response [12]. Two out of three patients who had Del19 mutation in primary tumors achieved a PR. Five out of nine patients who had WT K-ras in primary tumors achieved CR or PR. Two out of five patients who had mutant K-ras achieved a PR. Of the patients with mutant K-ras, one had WT EGFR and the response to EGFR TKI treatment was in a stable disease, and the others had mutant EGFR, and the response to EGFR TKI treatment was a PR (data not shown).

## 3. Discussion

Although lung cancer is still the leading cause of cancer death worldwide, the treatment of NSCLC has had fundamental changes in the past two decades. Substantial clinical benefits and improved overall survival rate have been achieved in patients with lung adenocarcinoma who are characterized and phenotyped carefully. With target therapy and chemotherapy on selected patients, the median overall survival time of advanced adenocarcinoma can be extended to more than 2.5 years [7].

Poor prognosis for lung cancer is mainly caused by the presence of a locally advanced or metastatic disease at diagnosis [27]. It has been reported that 10% to 36% lung cancer patients develop brain metastasis during the disease course [28,29]. Even in those patients who have already undergone curative treatment for primary tumor, there is still a high incidence of brain metastasis after surgery. Patients who developed brain metastasis were more likely to be adenocarcinoma, and most of them occurred early in the disease cause. A retrospective analysis of 646 patients who underwent surgery for lung cancer with curative intent reported a 6.3% incidence rate of postoperative brain metastasis, mostly occurring within 12 months of surgery [30].

At present, the greatest improvement in treating lung adenocarcinoma has been achieved with EGFR TKIs because EGFR mutations can predict the efficacy of such a treatment. In contrast, K-ras mutations are known as a useful biomarker of resistance to EGFR TKIs. There are several major signaling pathways mediating the downstream effects of EGFR activation, including the RAS/MAPK and PI3K/AKT pathways which are major signaling networks linking EGFR activation to cell survival and proliferation. Mutant K-ras has long been associated with primary resistance to EGFR inhibitors [31].

Different genotypes can exist within a primary tumor and its metastatic lesions as a heterogeneous complex. Such variations make anti-cancer therapy more challenging, especially in the era of target therapy, where the target may be lost or changed from one site to another. Although EGFR TKIs should be effective for tumors bearing active mutations, some patients still exhibit intrinsic resistance. The mechanisms for this resistance are largely unknown, although a minority of tumors may be related to T790M mutation or c-Met amplification, known to be involved in acquired resistance, in treatment-naïve specimens [32,33,34]. In our study, EGFR and K-ras changed from WT to mutant type and *vice versa* within the primary tumor and metastases in some patients. There were 26.5% patients had different genotypes in primary tumors and their brain metastases (Table 2). Therefore, the predictive power of EGFR mutations based on the primary tumor could be lessened, and rebiopsy from the metastatic sites, collecting circulating cancer cells or cell-free DNA for EGFR analysis should be considered, especially from the non-responsive sites. This discordance was even more prominent in K-ras, with a concordance rate of only 60.6%. More patients who had WT K-ras in the primary tumor had detectable mutant K-ras in the brain metastasis, which may possibly explain the different responses to therapy in primary tumors and metastatic sites (Table 3). In addition to the low concentration of metabolites of EGFR TKIs in brain leading to the lower response rate of brain metastasis [35], the high mutation rate of K-ras could also partially explain the resistance to EGFR TKIs in brain metastasis.

In a lung adenocarcinoma simultaneously harboring multiple heterogeneous clones of EGFR mutation and K-ras mutation, the effect of EGFR TKIs may be limited only to the parts carrying EGFR mutation but not to the other parts carrying K-ras mutations [36,37]. Because both EGFR and K-ras mutations are thought to be early events in lung adenocarcinoma [38] and K-ras mutations are closely related to smoking status [39], the reported coexistence of EGFR and K-ras mutations only accounts for about 5% of patients with EGFR mutations [40]. However, both EGFR and K-ras mutations can still exist simultaneously in multifocal adenocarcinomas of lung. Takamochi *et al.* reported coexisting EGFR and K-ras mutations in two (2%) of 82 patients with lung adenocarcinomas [36], and our result showed that there were 14 (28.6%) patients with both EGFR and K-ras mutations in the primary lung adenocarcinomas. Accordingly, combined EGFR and K-ras mutation analyses may be helpful in selection of treatment strategies for patients with lung adenocarcinomas. The K-ras mutation rate of our study was higher than previously reported, one possibility might be that we checked K-ras mutation by Sanger sequencing firstly, followed by real-time PCR for the cases that no mutation was detected by Sanger. It resulted in higher detection sensitivity for K-ras mutation. Another possibility might be related to the study cohort. We wonder if the cohort of lung adenocarcinoma with brain metastasis tends to have a higher frequency of double mutations. More data will be needed to answer this question. Recently, a review article also mentioned about the importance of highly sensitive and appropriately validated mutation analysis methodologies for better accuracy [6].

In our study, L858R mutation had a higher incidence of coexisting with K-ras mutation than Del19 mutation had (22.4% *vs.* 6.1%). In metastatic brain lesions, 12 (36.4%) patients had both EGFR and K-ras mutations synchronously, and again patients with L858R mutation had a higher incidence of K-ras mutations than those with Del19 mutation (27.3% *vs.* 9.1%, Table 4). The presence of combined mutations may explain why EGFR TKI treatment for patients with L858R has an inferior response, shorter response duration and worse overall survival [41]. On the other hand, the mutation status of K-ras was more variable compared with EGFR (Table 5), a higher percentage of tumors changing from WT to mutant type or *vice versa*. It might influence the availability to choose mutant K-ras as a therapeutic target.

A previous study reported that all tumors responded to gefitinib had WT K-ras [42], thus suggesting K-ras mutations are mutually exclusive with EGFR mutations [43]. However, two of our patients with mutant K-ras still achieved a PR after TKI treatment. We only had 14 patients receiving EGFR TKIs in this study, therefore it is difficult to draw conclusions about the relationships between the types of EGFR and K-ras mutations.

After the introduction of EGFR TKIs, there have been increased patients having solely progressive lesions in the central nervous system (CNS). Up to 33% of patients with EGFR-mutant lung cancer treated with EGFR TKIs will experience disease progression in the CNS [44]. Although EGFR TKIs are effective for mutation-positive patients, the concentration of the drug achievable in brain is only approximately 1% to 5% of the level found in the plasma [45,46]. Therefore, the mutation selection pressure could be different between peripheral organs and brain. In a previous study, the secondary EGFR T790M mutation was found in only four (13%) of 30 brain metastases, with a frequency far lower than that seen in peripheral organs [47,48]. Our results confirmed the genomic instability between primary lung adenocarcinoma and brain metastasis, and a higher co-existence of EGFR and K-ras mutations than that of previously reported, which may be one of the causes of intrinsic resistance. The absence of K-ras mutations does not guarantee an improved likelihood of a response to EGFR-targeting strategies in patients with NSCLC. However, somatic mutations leading to gain-of-function and constitutive signaling of the K-ras pathways may be predictive biomarkers for non-responsiveness to both monoclonal antibodies and TKI-based strategies [49].

## 4. Materials and Methods

### 4.1. Selection of Patients

Paired paraffin-embedded tissues of both primary adenocarcinoma of lung and matched brain metastases in the archives of the Department of Pathology in Kaohsiung Chang Gung Memorial Hospital from 1991 to 2010 were retrieved. Informed consents were obtained from patients who underwent surgical procedures after 2002 and were still alive when this study was conducted.

### 4.2. DNA Extraction

All the tumors were histologically reviewed to confirm the diagnosis and to select adequate areas for macrodissection for genomic DNA extraction. Five 10-μm sections of the paraffin-embedded primary lung tumor and brain metastasis specimens were sliced onto glass slides. After ensuring that the selected area contained more than 25% tumor cells, macrodissection was performed followed by deparaffinization. Genomic DNA was isolated using a Puregene Cell and Tissue Kit (QIAGEN Sciences, Germantown, MD, USA) or a PicoPureTM DNA Extraction kit (Arcturus Bioscience, Inc., Mountain View, CA, USA). If necessary, glycogen was added as a DNA carrier, and the final elution volume was reduced to a half in order to obtain more condensed DNA. The DNA concentration (ng/μL) and *A*_260_/*A*_280_ ratio were measured using a spectrophotometer (NanoVue, GE Healthcare, Pittsburgh, PA, USA). Fragments of DNA were also assessed with multiplex DNA internal controls, which included testing for five different DNA targets (100, 200, 300, 400 and 600 bp) in the same reaction.

### 4.3. K-ras Mutational Analyses by Direct DNA Sequencing

K-ras mutation was first detected by Sanger sequencing. The primers used for polymerase chain reaction (PCR) before sequencing were: forward (5′-AACCTTATGTGTGACATGTTC-3′) and reverse (5′-ATGGTCCTGCACCAGTAAT-3′). The PCR products were visualized on 2% agarose gels and archived prior to sequencing. Sense and antisense sequencing was performed in a 10-μL reaction with the BigDye Teminator v.1.1 Cycle Sequencing Kit (Applied Biosystems, Foster City, CA, USA) using an ABI3100A Capillary Genetic Analyzer (Applied Biosystems).

### 4.4. EGFR and K-ras Mutation Analysis

Detection of common EGFR mutations (Del19, L858R and L861Q in exon 21, G719X in exon 18, S768I in exon 20, and three insertions in exon 20) was performed using an EGFR Scorpion Amplification Refractory Mutation system (SARMS) (DxS Ltd., Manchester, UK) with real-time PCR reactions in an ABI 7500 Fast System (Applied Biosystems) according to the manufacturer’s instructions. Comparative threshold values were calculated using 7500 Fast System SDS Software. K-ras mutational analysis was performed using a Light Mix kit K-ras Codon 12/13 CE (TIB MOLBIOL, Berlin, Germany). Allele-specific real-time quantitative PCR was carried out using a Light Cycler 2.0 system (Roche Diagnostics Ltd., Taipei, Taiwan) according to the manufacturer’s instructions.

### 4.5. Ethical Approval

Approval to analyze and publish the aggregated anonymous data was given by the Institutional Review Board committee of Kaohsiung Chang Gung Memorial Hospital at Kaohsiung, Taiwan. The approval number of this project was 96-2865B. The approval date was on 20 October 2010.

## 5. Conclusions

To the best of our knowledge, this study recruited the largest case number with paired primary lung adenocarcinoma and brain metastasis for the analysis and comparison of EGFR and K-ras mutations. The limitations of this study include retrospective study design and lack of therapeutic responses of EGFR TKIs treatment for some patients due to tissue collection before the era of target therapy. In conclusion, the status of EGFR mutations in adenocarcinomas of the lung is relatively consistent between primary and metastatic sites compared to K-ras mutations. L858R had higher change to combine with mutant K-ras compared with Del19. However, some of the adenocarcinomas show a discordant status of EGFR mutations between the primary and metastatic sites. Accordingly, it is recommended to repeat analysis of EGFR mutation if tissue from metastatic or recurrent sites is available before the administration of target therapy or if the response of brain metastasis to EGFR TKI is not as expected. For patients who are not suitable for repeating biopsy, liquid biopsy either from circulating DNA or circulating tumor cells, is emerging as a powerful and convenient strategy for tumor genotyping [50].

## Figures and Tables

**Table 1:** Characteristics of the patients.

Characteristics	Number of Cases	%
All patients	49	100
Age at diagnosis	Years (range)	
Mean	64.0 (46–86)	
Median	63.0 (46–86)	
Sex		
Male	27	55.1
Female	22	44.9
Smoking history		
Never-smoker	18	36.7
Current or former smoker	26	53.1
Unknown	5	10.2
Stage at diagnosis		
I	3	6.1
II	4	8.1
III	3	6.1
IV	37	75.5
Not available	2	4.1
Brain metastasis		
Synchronous	31	63.3
Metachronous	18	36.7
EGFR TKI treatment		
Yes	14	28.6
No	35	71.4

EGFR, epidermal growth factor receptor; TKI, tyrosine kinase inhibitor.

**Table 2:** Result of EGFR mutation analysis.

Genotype of EGFR	Primary Tumor		Brain Metastasis		Same Genotype of Primary Tumor and Brain Metastasis	
	Case Number	%	Case Number	%	Case Number	% of Primary Tumors
Wild type	19	38.8	19	38.8	15	78.9
L858R only	17	34.7	13	26.5	13	76.5
Del19 only	10	20.4	15	30.6	8	80.0
L858R and Del19	3	6.1	2	4.1	0	0
Total	49	100	49	100	36	73.5

EGFR, epidermal growth factor receptor; Del19, deletion 19.

**Table 3:** Analysis of K-ras mutation.

Genotype of K-ras Mutation ^1^	Primary Tumor		Brain Metastasis		Same Genotype of Primary Tumor and Brain Metastases	
	Case Number	% ^2^	Case Number	% ^2^	Case Number	% of Primary Tumor
Wild type	19	57.6	17	51.5	12	73.7
Codon 12 only	2	6.1	2	6.1	2	100
Codon 13 only	4	12.1	2	6.1	2	50
Codon 12 & 13	8	24.2	12	36.4	4	50
Total	33	100	33	100	20	60.6

^1^ K-ras mutation of the tumors was checked by direct sequencing firstly, followed by allele-specific real-time quantitative PCR for cases without detectable K-ras mutation by direct sequencing; ^2^ %: the percentage of total 33 cases.

**Table 4:** Synchronous mutation status of EGFR and K-ras in primary lung adenocarcinomas and brain metastases.

EGFR /K-ras Status	Lung		Brain	
EGFR/K-ras ^1^	Case Number	%	Case Number	%
EGFR WT	19		11	
WT/WT	12	24.5	7	21.2
WT/Mut	7	14.3	4	12.1
L858R only	17		12	
L858R/WT	6	12.2	3	9.1
L858R/Mut	11	22.4	9	27.3
Del19 only	10		10	
Del19/WT	7	14.3	7	21.2
Del19/Mut	3	6.1	3	9.1
Others	3		0	0
Del19 + L858R/WT	3	6.1	0	0
Total	49	100	33 ^2^	100

EGFR, epidermal growth factor receptor; WT, wild type; Del19, deletion 19; Mut, mutation of K-ras, including codon 12, codon 13 or codon 12 and 13; ^1^ There were 28 primary lung adenocarcinomas and 17 brain metastases with wild type K-ras; ^2^ There were only 33 paired brain metastases with available samples for checking K-ras mutation status.

**Table 5:** Mutation shift between primary tumors and brain metastases.

	Primary Tumor	Brain Mets		Primary Tumor	Brain Mets	
	WT	Mut	Discordant Rate	Mut	WT	Discordant Rate
EGFR	19	4	21%	30	4	13.3%
K-ras	19	7	36.8%	14	5	35.7%

EGFR, epidermal growth factor receptor; WT, wild type; Mut, Mutant; Mets, metastasis.

**Table 6:** Therapeutic response of primary tumors and corresponding mutation status of EGFR and K-ras of 14 patients.

Gene	CR (*n*)	PR (*n*)	SD (*n*)	PD (*n*)	NA (*n*)	Total
EGFR						
Wild type	1	0	1	2	1	5
L858R	0	4	0	2	0	6
Del19	0	2	0	0	1	3
Total	1	6	1	4	2	14
K-ras						
Wild type	1	4	0	2	2	9
Mutation	0	2	1	2	0	5
Total	1	6	1	4	2	14

EGFR, epidermal growth factor receptor; CR, complete response; PR, partial response; SD, stable disease; PD, progressive disease; NA, not available.

## Abbreviations

NSCLC: non-small cell lung carcinoma; EGFR: epidermal growth factor receptor; TKIs: tyrosine kinase inhibitors; Del19: exon 19 deletion; L858R: exon 21 point mutation; PCR: polymerase chain reaction; WT: wild type; CR: complete response; PR: partial response.
